# Repeated mild traumatic brain injury can cause acute neurologic impairment without overt structural damage in juvenile rats

**DOI:** 10.1371/journal.pone.0197187

**Published:** 2018-05-08

**Authors:** Alicia Meconi, Ryan C. Wortman, David K. Wright, Katie J. Neale, Melissa Clarkson, Sandy R. Shultz, Brian R. Christie

**Affiliations:** 1 Division of Medical Sciences, University of Victoria, Victoria, British Columbia, Canada; 2 The Florey Institute of Neuroscience and Mental Health, Parkville, Victoria, Australia; 3 Department of Neuroscience, Monash University, Melbourne, Victoria, Australia; 4 Department of Medicine, University of Melbourne, Parkville, Victoria, Australia; 5 Centre for Brain Health and Program in Neuroscience, University of British Columbia, Vancouver, British Columbia, Canada; 6 Department of Cellular and Physiological Sciences, University of British Columbia, Vancouver, British Columbia, Canada; Cleveland Clinic, UNITED STATES

## Abstract

Repeated concussion is becoming increasingly recognized as a serious public health concern around the world. Moreover, there is a greater awareness amongst health professionals of the potential for repeated pediatric concussions to detrimentally alter the structure and function of the developing brain. To better study this issue, we developed an awake closed head injury (ACHI) model that enabled repeated concussions to be performed reliably and reproducibly in juvenile rats. A neurological assessment protocol (NAP) score was generated immediately after each ACHI to help quantify the cumulative effects of repeated injury on level of consciousness, and basic motor and reflexive capacity. Here we show that we can produce a repeated ACHI (4 impacts in two days) in both male and female juvenile rats without significant mortality or pain. We show that both single and repeated injuries produce acute neurological deficits resembling clinical concussion symptoms that can be quantified using the NAP score. Behavioural analyses indicate repeated ACHI acutely impaired spatial memory in the Barnes maze, and an interesting sex effect was revealed as memory impairment correlated moderately with poorer NAP score performance in a subset of females. These cognitive impairments occurred in the absence of motor impairments on the Rotarod, or emotional changes in the open field and elevated plus mazes. Cresyl violet histology and structural magnetic resonance imaging (MRI) indicated that repeated ACHI did not produce significant structural damage. MRI also confirmed there was no volumetric loss in the cortex, hippocampus, or corpus callosum of animals at 1 or 7 days post-ACHI. Together these data indicate that the ACHI model can provide a reliable, high throughput means to study the effects of concussions in juvenile rats.

## Introduction

Concussion is a term used to clinically define the immediate and transient symptoms of a mild traumatic brain injury (mTBI)[1]. There are an estimated 1.6–3.8 million sports-related concussions per year in the USA alone [2–4], and this is a small proportion of the total number of concussions when non-sport related falls, vehicular accidents, and assaults are taken into consideration [5,6]. Concussions occur when a blow to the head or body causes a rapid movement of the brain in the skull. They only cause loss of consciousness (LOC) in a small fraction of cases [7–10], and usually do not involve skull fracture or significant bleeding in the brain; signs of more severe traumatic brain injury [5,11]. Instead, concussions are thought to result from microscopic damage and metabolic changes that manifest as a variety of symptoms including impaired memory, anxiety, balance and motor deficits, impaired vision, confusion, focus and attention deficits, dizziness, nausea, sleep disturbance, and headache [5,6,10,12]. Diagnoses can be difficult for physicians, as concussions typically cannot be detected with standard neuroimaging scans like computed tomography (CT) or MRI [5,13]. Instead, concussions are diagnosed by an assessment of observed signs and self-reported symptoms, and quantified with tools like the Glasgow coma scale or the sport concussion assessment tool (SCAT) [5,11,14].

Although concussions are defined as a mild brain injury, the daily activities of those affected can be greatly disrupted. The primary treatment for a concussion is usually physical and cognitive rest until symptoms resolve spontaneously within 7 to 10 days [5]. In a subset of those injured, symptoms can persist for months and years, often called post-concussive syndrome [15,16]. Clinical research has identified age as one of several important risk factors that can predispose an individual to post-concussive syndrome, as children and adolescents may be more likely to develop persistent symptoms [17–19]. Prior mTBI events put the patient at greater risk of sustaining additional head injuries, known as repeated mTBI [20–22]. Patients with a history of repeated mTBI display increased learning and memory impairment [23–25], slowed balance recovery [26], impaired visuospatial perception [24], difficulty in concentration, and increased incidence of headaches [27]. While symptoms of a single injury resolve spontaneously, repeated injuries may cause symptoms to persist for extended periods [28–30]. Moreover, increasing evidence suggests a link between repeated mTBI and increased risk of developing dementia [31] and other neurodegenerative diseases [32,33]. In particular, more work is needed to understand how the developing brain is uniquely vulnerable to concussion, and the long term impact(s) of repeated mTBI.

Animal models of traumatic brain injury (TBI) are an important tool to help understand the pathophysiology of concussions, and for developing diagnostic and treatment strategies [34]. Several animal models have been developed to study TBI, and they have been instrumental in understanding how the brain reacts to trauma [35,36]. While these models have provided the basis for a growing understanding of the complex neurometabolic changes that accompany TBI [37], it is important to acknowledge that technical aspects of many models, such as the surgical disruption of the skull and the use of anaesthesia, [38–40] may limit how these models can be used to understand the unique pathophysiology that results from mild closed head injuries. Furthermore, current models focus disproportionately on single incidents in adult subjects rather than repeated mTBI in the juvenile population, which is normally a population that is more at risk for sports related concussions.

To address the critical need for animal models that accurately mimic concussions in juveniles, we have adapted a model used to produce mild closed head injuries in adult mice without anaesthesia [41,42] for use in juvenile rats. Here we show that mTBIs with the awake closed head injury (**ACHI**) model produces deficits similar to those observed in clinical cases of concussion. The ACHI model will be a useful tool in our efforts to understand how concussions affect the developing brain.

## Materials and methods

### Subjects

All procedures used in this study were approved by the University of Victoria Animal Care Committee and are in compliance with Canadian Council for Animal Care guidelines. Juvenile Long Evans rats (n = 94) were obtained from (Charles River Laboratories, St. Constant, PQ) or bred at the University of Victoria. Offspring were weaned at postnatal day (PND) 21 and housed in same-sex groups of 2–3. They were then assigned to one of three experimental groups (sham control, single ACHI, or repeated ACHI) so that no more than two subjects from any one litter were assigned to any one experimental group. Average weight at the time of the first procedure was 70.0 g for males and 66.1 g for females. All subjects were housed under standard laboratory conditions including automatically controlled temperature, humidity, ventilation and a 12-hour light/dark cycle with *ad libitum* food and water access. All purchased animals were allowed to adapt to the vivarium for at least one week prior to experimental procedures. After injury or behavioural testing animals were returned to their home cages unless otherwise specified.

### ACHI procedure

**Fig 1:**
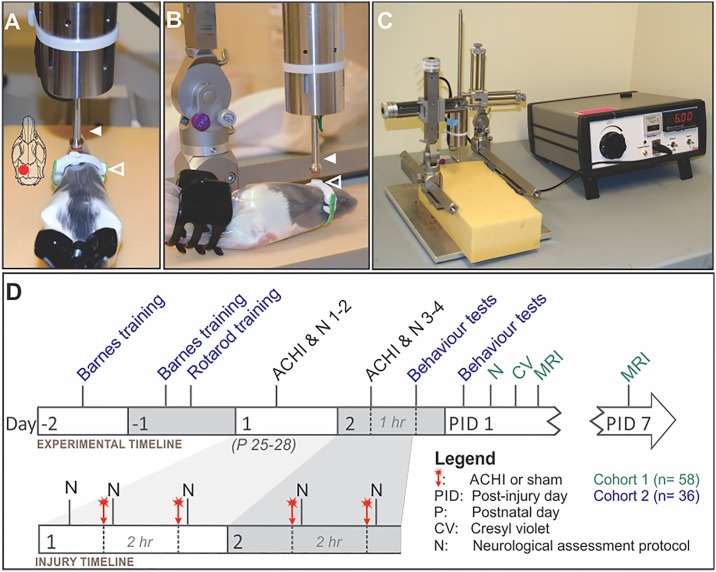
**(A-C)** Awake closed head injury (ACHI) apparatus and **(D)** experimental timeline. **(A, B)** Subjects were placed in a soft plastic restraint bag on a foam platform. A custom 3D printed helmet (open arrowhead) was placed on the head, with the impact site centered over the left parietal cortex. **(C)** A modified Leica Impact One controlled cortical impactor was used to generate the injury. It was modified by adding a 7 mm diameter rubber impact tip (closed arrowhead), and was set to a velocity of 6 m/s and dwell time of 100 ms. The impactor was centered over the helmet target and adjusted to depress a depth of 10 mm, and the control box was used to initiate the impact. Animals in the sham control group underwent the exact same procedure, except the impactor was deployed beside their head. **(D)** Two cohorts of male and female juvenile Long Evans rats underwent two separate sets of experiments. Animals in cohort 1 were used for neurologic severity scoring, MRI analysis, and cresyl violet histology. Animals in cohort 2 were trained for the Barnes maze and Rotarod in the two days immediately before ACHI, and then were tested in the Barnes maze, Rotarod, elevated plus maze, and open field maze at one hour and one day after the final ACHI. Both cohorts followed the same injury timeline: Animals in the repeat ACHI group received two impacts per day, with a two-hour interval between injuries, for two days. The single ACHI group received three sham procedures followed by one impact, on the same timeline as the repeat ACHI group. Sham control animals received four sham procedures on the same timeline as the other groups. The NAP was performed immediately after each ACHI or sham procedure on all subjects, and a subset from cohort 1 were tested again 24 hours later.

We developed the ACHI model to produce a mild closed head injury in juvenile rats without the use of anaesthesia. It is adapted from procedures reported in adult mice [41,42]. As shown in Fig 1, beginning on PND 25–28, subjects were immobilized for the procedure using clear plastic restraint cones (Model DC-200, Braintree Scientific, Braintree, MA). The cones have an opening at the nostril to provide ventilation, and are held closed behind the haunches using a plastic hair clip **(**Fig 1A and 1B). Custom 3D printed plastic helmets were used to help dissipate the force of the blow across the skull and reduce the chance of skull fracture **(Open arrows**, Fig 1A and 1B; Schematic is shown in S1 Fig; Replicator-2, MakerBot, Brooklyn, NY; 1.75 mm ABS plastic filament). The helmets were held in place with an elastic band and double-sided tape. The back of the helmet was aligned with the interaural line, and a flat circular surface (7 mm diameter) on the top of the helmet aided in targeting the impact over the left parietal cortex (Fig 1A). A modified controlled cortical impact device (Impact One, Leica Biosystems Inc., ON, Canada) was mounted on a stereotaxic frame. The impactor was modified with the addition of a 7 mm diameter flat rubber tip (**Closed arrows**, Fig 1A and 1B). The rats were placed on a soft foam platform (3” thick Super-Cushioning Polyurethane Foam Sheet, McMaster-Carr, OH) directly below the impactor. The impactor tip was carefully targeted over the left parietal cortex, and an electromagnetic piston drove the impact tip into the helmet at a speed of 6 m/s, and depth of 10 mm. The impactor was retracted immediately (100 ms dwell time) to prevent ricochet. After each impact subjects were immediately removed from the restraint bag for assessment.

Subjects in the repeated ACHI group received two impacts per day over two consecutive days for a total of four impacts (Fig 1D). On each day the impacts occurred at the same time, and there was a two-hour interval between impacts. Subjects in the sham group received four sham procedures on the same schedule. In the sham procedure, they were restrained and prepared for impact in a manner identical to the ACHI groups, but the impact tip was deployed beside their head instead of hitting it. Subjects in the single ACHI group received three sham procedures and then one impact on the same schedule.

### Restraint tolerance

During all ACHI or sham procedures animals were assessed for tolerance of the restraint cone. Animals were scored on a scale of 0–4 (S1 Table) based on willingness to enter the cone, and on movement and vocalisation while restrained. Any animal that reached a score of 4 or greater (e.g. >10 instances of vocalisation or movement; restrained in cone for 5 minutes) was immediately removed from the restraint and returned to the home cage for 15 minutes minimum. Protocol required that the procedure be re-attempted up to three times, and if it could not be completed the animal be removed from the study, however no animals were removed from the current study.

### Pain assessment

All animals were assessed immediately before, and for several days after, each ACHI for indications of pain or discomfort that included changes in locomotion (i.e. immobility, slowness of movement), behaviour (i.e. hunched posture, piloerection, excessive stretching, teeth grinding) pain on palpitation of the impact site (i.e. vocalization, withdrawal of head), skin turgor (i.e. increased tenting), and changes in weight relative to age and sex-matched littermates (decreased by more than 5%). Scoring scales developed with our institutional Animal Care Committee are shown in S2 Table). Animals were rated in each of the 5 categories on a scale of 0 (normal) to 3 (severe) with a score of greater than 2 in any category, or a combined score of greater than 3, requiring the animal be given supportive care. Animals with a score of 3 in any category or a combined score of greater than 6 were to be removed from the study and euthanized.

### Level of consciousness

Three common tests were performed immediately after each procedure to provide a convergent assessment of the animal’s level of consciousness.

1. **Apnea**
  After being removed from the restraint bag and placed upright on a clean surface each subject was initially examined for apnea. If they were not breathing, the amount of time from the start of the test until breathing returned was recorded as the latency to recovery.
2. **Toe Pinch Reflex**
  The toe pinch reflex was then assessed by gently extending the subject’s contralateral (to injury hemisphere) hind limb and pinching sharply and firmly. If the subject did not immediately retract the limb, the pinch was repeated at five second intervals on alternating hind limbs. The time from the first pinch until the subject retracted their limb was recorded as latency to recovery.
3. **Righting Reflex**
  The righting reflex was determined by placing the subject on their back. The subject should immediately flip themselves upright, and if they did not the amount of time taken for the subject to right themselves was recorded.

### Neurological assessment protocol

The ACHI model uniquely allowed us to immediately perform a neurological assessment after each impact, without being delayed or affected by recovery from anesthesia. Similar to that of others, [43–46] our NAP score assessed four basic neurological outcomes after each procedure. It consists of four simple reflexive and motor tasks, which can all be assessed within the first minute after the ACHI or sham. Each task is scored simply as a pass or fail, and a point is awarded for each pass. A score of four indicates perfect performance and a NAP score of zero indicates all four tasks were failed. The components of the NAP are as follows:

1. **Startle Reflex**
  The subject is placed in the center of a clean, empty, standard housing cage, and the researcher claps loudly above the center of the cage. A point is given if the subject exhibits a startle response to the hand-clap. (Panel A in S2 Fig)
2. **Limb Extension**
  The subject is grasped by the base of the tail and raised 30–50 cm in the air to examine the limb extension response. A point is given if both forelimbs are fully extended in response, and not if one or both are contracted or immobile (Panel B in S2 Fig).
3. **Balance Beam**
  The animal is placed on a flat narrow balance beam and their ability to balance and walk is assessed. The beam itself is 100 cm long x 2 cm wide x 0.75 cm thick, and is placed 22 cm above a cushioned work surface that extends from an empty cage to the animal’s home cage. The subject is placed squarely balanced on the center of the beam, facing the home cage. A point is given if they are able to walk on the beam using all four limbs. Animals that are immobile, unable to grasp the beam with any limb, or that fall from the beam are given a failing grade (Panel C in S2 Fig).
4. **Rotating Beam**
  The subject’s ability to navigate a slowly rotating beam is assessed. The subject is placed squarely balanced on the center of the beam used in the previous task at a height of 75 cm above a cushioned work surface, and the beam is rotated once per second for 4 rotations. A point is scored if the subject remains on the rotating beam for the complete duration, and not if they fall (Panel D in S2 Fig).

### Cresyl violet stain & imaging

To determine if the ACHI procedure caused significant anatomical damage, a cohort of 6 animals were euthanized with isoflurane and then perfused with heparinized saline followed by 2% paraformaldehyde. Brains were sectioned at 50 μm and regions of interest were mounted on gelatinized slides before being stained with 0.1% aqueous cresyl violet (Sigma, St. Louis, MO), dehydrated in ethanol, cleared in xylene and cover-slipped with Permount mounting medium (Fisher Scientific, PA, USA).

### MRI acquisition & analysis

To further examine structural damage in the ACHI model we performed *ex vivo* structural MRI on both male and female cohorts of sham, single- or repeated ACHI rats at one and seven days after injury. The number of brains scanned for each of the groups were: sham, 1 day = 8 (3 males, 5 females); sham, 7 days = 9 (4 males, 5 females); single mTBI, 1 day = 11 (4 males, 7 females); single mTBI, 7 days = 7 (5 males, 2 females); repeated mTBI, 1 day = 11 (5 males, 6 females); repeated mTBI, 7 days = 7 (3 males, 4 females). Animals were perfused as described above, and after the brains were removed, they were embedded in agar gel (Webster et al., 2015) and scanned using a 4.7 Tesla Bruker Advance III MRI fitted with a BGA12S2 actively shielded gradient set. Actively decoupled volume transmit and 4-channel surface receive coils (Bruker, Germany) were used to acquire a multi-echo, T_2_*-weighted image with the following imaging parameters: repetition time = 8 s; 12 echoes with the first echo at 15 ms and an echo spacing of 7.5 ms; field of view = 2.304 × 2.048 mm^2^; matrix size = 144 × 128; resolution = 160 × 160 μm^3^; number of slices = 74; slice thickness = 160 μm; and number of excitations = 2.

Spatial intensity in homogeneity in T_2_*-weighted images was corrected by estimating the bias field with N4 Bias Correction [47] and template images generated for sham, single and repeated injury cohorts at each time point using Advanced Normalization Tools (ANTs, http://stnava.github.io/ANTs/)[48]. The resulting template images were then combined using ANTs into a study-specific template [49,50] that was segmented into different tissue classes using FAST [51]. The FAST segmentations were used to guide the tracing of six *a-priori* regions of interest (ROIs) including the ipsilateral and contralateral cortex, corpus callosum and hippocampus [52–54]. The ROIs were registered to subject space using inverse subject-to-template diffeomorphisms and the total volumes for each structure were calculated using FSL stats, a component of FMRIB’s Software Library (FSL, http://www.fmrib.ox.ac.uk/fsl). MRI analysis was conducted by a researcher who was blinded to the experimental conditions.

### Behavioral assessment

In addition to the NAP, four behavioural tasks were employed to examine the behavioral sequelae to repeated ACHI in both male and female animals. A cohort of repeated ACHI (n = 9 female, 9 male) and sham injured subjects (n = 9 female, 9 male) were assessed using the following behavioural tasks at one hour and one day after final injury:

#### Barnes maze

The Barnes maze was used to assess spatial memory after injury. The Barnes maze (Maze Engineers, Cambridge, MA) is an elevated circular platform (122 cm diameter) with 20 holes (10cm diameter) spaced evenly around the perimeter. One hole leads to an escape box that remains in the same position throughout the experiment, and the other holes are open. The maze was placed in a room with distinct distal visual cues to allow for spatial navigation. The training paradigm used was adapted from common procedures in order to suit the timeline of this experiment and the juvenile age group [55]. All subjects were trained to locate the escape hole for two days before ACHI or sham. The first training day consisted of 4 trials, and on the second training day trials repeated until criterion was reached. The training criterion was determined as the ability to locate the escape hole using a direct search method with ≤ 1 error in two trials. Immediately before the first training trial rats were enclosed in the escape box to acclimatize for 2 minutes. Then they were moved directly to the center of the maze and allowed to explore freely for 5 minutes. If they found the escape box during this time, they remained there for 15 seconds before being returned to their home cage. If they did not find the escape box, they were led there by the researcher and allowed to remain there for 15s before being returned to their home cage. All remaining training trials were the same as the first, except the acclimatization period was not repeated, and instead subjects were placed in the center of the maze directly from their home cage at the start of the trial. A test trial was completed one hour and one day after injury. Like the training trials, rats were placed in the center of the maze and allowed to explore freely for up to 5 minutes. The total distance travelled and number of errors made before locating the escape hole was measured as an indication of spatial memory. Movement in the maze was tracked using EthoVision XT 11.5 software (Noldus, Netherlands). Error tracking was performed manually by a researcher blinded to group. An error was scored if the subject moved any portion of their head over a hole that did not allow escape. All maze components were wiped down with Virkon disinfectant/cleaner and allowed to dry completely between subjects.

#### Rotarod

Motor coordination and balance were assessed using the Rotarod (Rat Rotarod NG, Model 47750; Ugo Basile, Varese, Italy). The apparatus consists of a rotating rod (6 cm diameter) with machined grips, divided into four equal 8.7 cm wide sections raised 30 cm above trip boxes. Subjects were trained to use the Rotarod one day before the first ACHI or sham. In training trials, subjects were placed on the rod, which was rotating at a constant speed of 10 rpm. The training trial continued until the subject able to stay on the rod for 60 consecutive seconds without falling, turning around, or clinging to the rod. If they fell from the rod or turned around, they were placed back on the rod correctly and the timer restarted. In test trials, an accelerating protocol was used with the speed of rotation increased from 10–50 rpm over 300 s. Each trial was terminated if an animal fell, clung and rotated for two full rotations, or remained on for >300s. Latency to fall (s) were automatically recorded for each trial. The average of the three trials was calculated and used for analysis. Training trials and baseline values were recorded 24 h prior to ACHI procedure. The Rotarod apparatus was wiped down with Virkon and allowed to dry completely between subjects.

#### Open field

The open field was used to assess anxiety-like behaviour, and overall locomotion. Animals were placed in the center of a circular white, arena (100 cm diameter, 50cm walls in a brightly lit room and given 5 min to explore freely [53,56,57]. Animals were tracked with EthoVision XT 11.5 software (Noldus, Netherlands). Increased time spent in the perimeter (thigmotaxis) or decreased time spent in the center area (70 cm diameter) are measures of anxiety-like behaviour [58,59]. Secondary measures included proportion of time moving and average velocity of movement. The maze was wiped down with Virkon and allowed to dry completely between subjects.

#### Elevated plus maze

Anxiety like behaviour was also assessed in the elevated plus maze after ACHI [60,61]. A raised plus-shaped maze with two opposing enclosed arms and two opposing open arms in a brightly lit room was used. Rats were placed in the center of the maze facing a closed arm, and allowed to explore freely for 5 minutes. Animals were tracked using EthoVision XT 11.5 software (Noldus, Netherlands). The proportion of time spent in the open arms was measured as an indication of anxiety. Secondary measurements taken were proportion of time moving, and average velocity. The maze was wiped down with Virkon and allowed to dry completely between subjects.

#### Statistical analysis

A Kruskal-Wallis test with Nemenyi *post hoc* analysis were used to compare the composite NAP, as this is a non-parametric dataset. Two-way ANOVAs with injury group and post-injury time point as the between subject factors were used to analyze MRI volumetrics for each ROI. For Barnes maze training trials, mixed ANOVA with trial number as within subject factor and sex as between subjects factor was used to analyze individual trial path lengths. A Greenhouse-Geisser correction was used to adjust for violation of Mauchly’s test of Sphericity. Two-way ANOVA with injury group and sex as between subject factors was used to analyze total path distance. Mixed ANOVA with injury group and sex as between subjects factors, and post injury time point as the within subjects factor were used to analyze behavioural task outcomes. Statistical significance was set at p<0.05. Linear trendlines and correlation coefficients in the comparison of NAP scores to Barnes maze outcomes were determined using Excel (Microsoft, Redmond, WA). Power analyses were performed using G*-Power to determine group sizes required for appropriate statistical analyses. Statistical analyses were performed using RStudio (RStudio, Boston, MA) and SPSS statistics software (IBM, New York, NY).

## Results

### ACHI model demographics

The animals’ welfare and comfort were prioritized by taking extra care to actively monitor and respond to changes in each animal’s tolerance of the restraint, and signs of pain post injury.

#### Restraint tolerance

Animals were assessed for restraint tolerance according to a predetermined scoring index approved by our institutional Animal Care Committee. Overall the subjects tolerated the restraint well. Common indications of low tolerance were mild vocalisation, and shifting position in the restraint. The ACHI was only performed if the subject was motionless for the impact component and was not vocalizing during the procedure. In the current cohort (n = 97), there were no instances where a subject had to be removed from the study due to restraint intolerance. There was one instance (repeated ACHI male, 4^th^ injury) where the subject was returned to their home cage due to persistent movement, but the procedure was successful on the second attempt.

#### Pain assessment

Subjects were regularly assessed for signs of pain, and scored according to a predetermined index throughout the experiment and following days. Subjects showed almost no signs of pain across all criteria, and no animals received a combined pain scale score of greater than 2, so supportive care and/or analgesics were not required for any subjects in these test populations.

#### Mortality

The overall mortality rate for this model was relatively low at 3% (3/97). These cases were all males from the repeated ACHI group, following their fourth ACHI procedure. In all three cases, it was immediately obvious that the subject was moribund, so they were immediately euthanized and removed from the study. There were no cases where a subject initially appeared normal, and then escalated to a moribund state.

These data indicate that the ACHI procedure is a simple and high throughput method of producing a mild closed head injury. The ACHI procedure was consistently completed, including the NAP, in less than 5 minutes per animal.

### Quantification of mild neurological impairments in ACHI

#### Loss of consciousness

**Fig 2:**
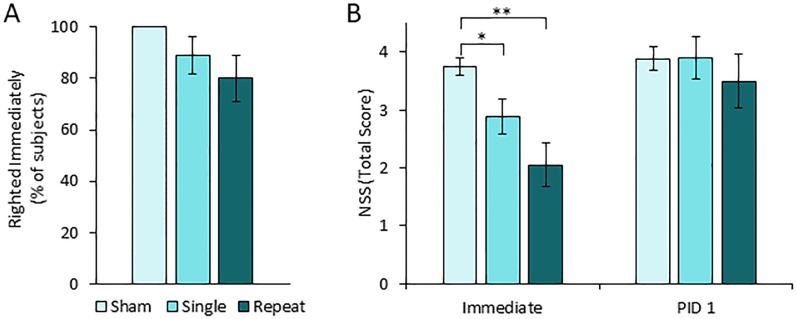
Awake closed head injury produces short-lived loss of consciousness and acute neurologic impairment without producing a visible lesion. (**A**) Righting reflex was assessed immediately after ACHI or sham. 100% of sham subjects self-righted immediately, whereas 11% of single ACHI, and 20% of repeat ACHI subjects failed to right immediately, indicating an acute loss of consciousness. Neurologic assessment protocol (NAP) testing began immediately after the righting reflex test, and was repeated one day later in a subset of animals. They were assessed in the startle reflex, limb extension, beam walk, and rotating beam tasks, and given a point for each successfully completed task so that a score of 4 indicates perfect performance, and a score of 0 indicates severe impairment. (**B**) Immediately after injury or sham the average NAP score was significantly lower in single and repeat ACHI groups compared to sham. (**B**) 1 day after ACHI or sham there were no significant differences in NAP scores.

We were able to assess three indicators of loss of consciousness (LOC) (apnea, toe pinch reflex, and righting reflex) immediately after each ACHI or sham procedure, without the potential confound of recovery from anaesthesia. As expected, subjects in the sham group did not exhibit any indication of LOC for any of the three indicators (Fig 2A). Apnea was not observed following the ACHI procedure in any of the 93 subjects in any of the conditions (sham, single, repeat). The toe pinch reflex was absent in one animal from the repeated ACHI group for 5 seconds. The righting reflex was briefly impaired in 11% of single ACHI animals and 20% of repeated ACHI animals. For these animals, the average latency until the righting reflex recovered was 4 seconds in the single ACHI group and 21.75 seconds in the repeated ACHI group. Chi square analysis indicate there are no significant differences in the proportion of animals from each group that failed toe-pinch (χ^2^(2, N = 58) = 1.93, p = 0.38) and righting reflex tests (χ^2^(2, N = 58) = 4.33, p = 0.11).

#### Neurological assessment protocol

NAP score analysis was completed in a cohort of 58 subjects immediately after each ACHI or sham (n = 18–20 per group), and in a subset of these it was repeated 24 hours later (n = 8–11 per group). The average number of NAP tasks successfully completed by each animal was quantified for each group and presented as a composite score, where a score of 4 indicates perfect performance and 0 indicates severe impairment. Immediately after the procedure, average NAP scores for the sham, single ACHI, and repeated ACHI groups were 3.8, 2.9, and 2.1 out of 4 respectively (Fig 2A). Kruskal-Wallis analysis revealed a significant effect of injury group (χ^2^ (2) = 19.35, p = 0.000063), and Nemenyi *post hoc* analysis showed that both single (p<0.05) and repeated ACHI (p<0.001) groups had significantly lower scores than sham animals. One day after the final ACHI, there were no significant differences between any of the treatment groups in NAP scores (χ^2^ (2) = 5.25, p = 0.07) (Fig 2B).

Since this is a new condensed format of components routinely performed in other common neurological assessments[42,45,46,62] we also assessed each task individually in order to determine how effectively they discriminate between injury groups. The success rates for each task (i.e. the percentage of subjects to pass each task) are shown in S2 Fig. In the startle response and limb extension tasks, 100% of shams passed, whereas only 55% and 40% of repeat injured subjects passed, respectively. The beam walk and rotating beam tasks appeared to be more challenging, as the shams had 90% and 85% success rates, respectively, indicating a subset of uninjured subjects failed this task as well. The beam walk still differentiated between experimental groups, as only 45% of repeat injured subjects passed by comparison. The rotating beam task may have been less effective for discriminating between groups, as 65% of repeat injured subjects passed.

### ACHI does not result in volumetric loss of cortex, hippocampus and corpus callosum

**Fig 3:**
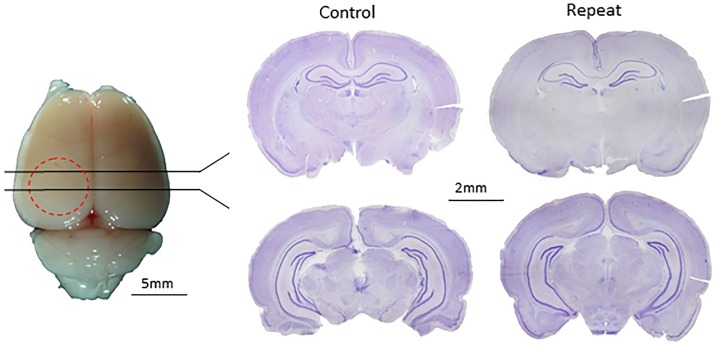
Repeat ACHI does not produce visible morphological abnormalities in the cortical layers beneath the injury site. Representative images of a perfused and formalin fixed brain from a female rat 24 hours after repeat ACHI (left), and 50 μm coronal slices from control and repeat ACHI subjects, stained with Cresyl violet (right). Low magnification images (20x) are shown and the injury side is the left in each image. (PID: Post injury day, *p<0.05, **p<0.01).

**Fig 4:**
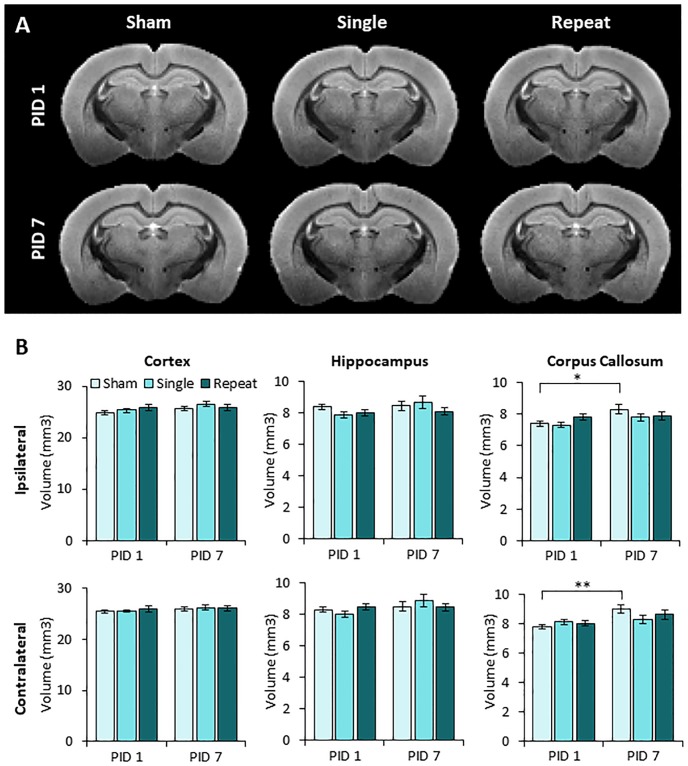
Single and repeat ACHI did not lead to overt brain damage observed in *ex vivo* structural MRIs in juvenile male and female rats. **(A)** Representative coronal slices of MRI scans at one and seven days following sham, single, or repeat ACHI show no obvious damage. **(B)** The volume of the ipsilateral and contralateral cortex, hippocampus, and corpus callosum were measured at PID1 or PID7. There was no significant effect of injury, or injury and recovery interaction in each of the six ROI’s. There was a significant effect for recovery time on volumes of the ipsilateral and contralateral corpus callosum. (* p<0.5; ** p < .01; PID: Post injury day).

Significant structural damage is unusual in clinical cases of concussion[18], but common in many rodent models of traumatic brain injury where the brain is directly impacted (e.g.,[50]). To determine if the ACHI model induced significant structural damage, we initially examined 50 μm brain sections from a cohort of 6 animals. As is illustrated in Fig 3, sections obtained from animals perfused and fixed 24 hour following their fourth ACHI procedure did not show evidence of severe tissue damage or hemorrhage. To examine brains in greater detail, structural MRI was performed on a separate cohort of animals to determine if the ACHI procedure led to volumetric changes in key structures. As shown in Fig 4A, no significant regions of damage could be identified with the structural MRIs performed on the brains of animals that received either the single or repeated ACHI procedure. This observation was supported by the lack of a significant injury or recovery time effect or interaction on volumetric measures from the six ROIs (Fig 4B).

### ACHI produces mild acute cognitive impairment in juvenile female rats

**Fig 5:**
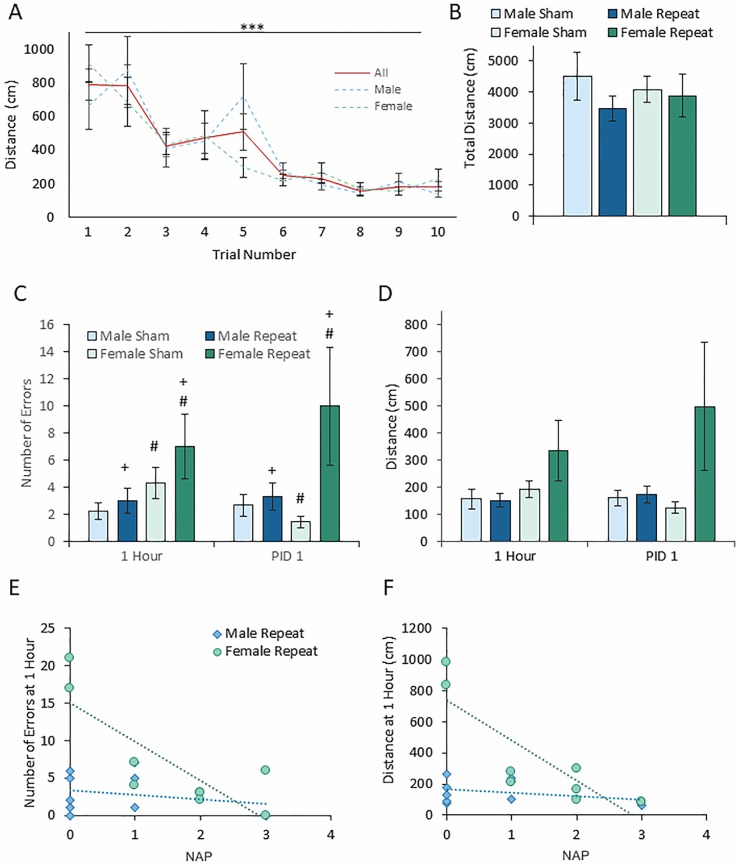
Repeat ACHI did not produce significant cognitive deficits in the Barnes maze compared to sham at one hour or one day after final injury. (**A**) Subjects were trained to use distal spatial cues to locate the escape hole in a Barnes maze in a series of 5 minute training trials over two days. The predetermined training criterion was to locate the hole using a direct search strategy with 1 or fewer errors in two trials. Subjects were able to reach the training criterion within an average of ten trials. There was a significant effect of trial number, but no significant interaction with sex. (**B**) The total distance travelled during training trials was equivalent between groups, indicating that all groups were equally proficient in the task before ACHI or sham. (**C**) There were no significant differences in the total distance travelled to escape during test trials. (**D**) There were no significant interactions of injury, sex, and/or post injury time point, but a significant effect of group indicates that across time points and sexes, the repeat ACHI group made more errors. Similarly, a significant effect of sex indicates that across injury groups, females made more errors than males. A subtle trend towards a longer distance to escape accompanied by elevated variability indicate that a subset of subjects in the repeat ACHI group were impaired. (**E-F**) This was further explored by plotting the distance to escape versus individual NAP scores for repeat ACHI subjects, in order to determine whether NAP score performance predicted cognitive outcomes. (**E**) A moderate negative correlation was observed in the female group between the NAP score immediately after injury, and the distance taken to escape the Barnes maze at one hour (R^2^ = 0.74). This was not seen in the males, and (**F**) had subsided in the females at one day (R^2^ <0.1). (PID: Post-injury day; *** p<0.001; + main effect of group, p<0.05; # main effect of sex, p<0.05).

Memory deficits are a common cognitive symptom of concussion in clinical populations [5,18] and rodent models alike[50,63–65], and in these experiments we used the Barnes maze to determine whether our ACHI model impairs recall memory. Subjects were trained to locate a small escape hole on an open elevated circular platform using distal spatial cues (n = 9 per group) prior to undergoing the ACHI procedure. After the ACHI procedure, their ability to remember the location of the escape hole was assessed. There was a significant effect of time (F (4.402, 149.680) = 14.681, p<0.001) but no interaction with sex across training trails, indicating that all subjects were able to learn the location of the escape hole (Fig 5A). On average this required 10 training trials). After randomly assigning subjects to injury groups, no differences were found in the total distance travelled during training (Fig 5B) confirming that groups were equally proficient in the task before injury. The total number of errors, and total distance taken to locate the Barnes maze escape hole were measured one hour and one day after ACHI. As shown in Fig 5C, a significant main effect of injury group (F(1, 32) = 5.857, p = 0.021), indicated that across both sexes and both time points, the animals from the repeated ACHI group made more errors during testing. A significant main effect of sex (F(1, 32) = 4.874, p = 0.035), indicates that across injury groups and time points, females made more errors than males. There were no significant effects of time point, or interactions between factors. As shown in Fig 5D There were no significant interactions or effects on the total distance to locate the escape hole, although a main effect of group approached significance (F(1,32) = 4.156, **p = 0.050**) with a moderate effect size (η_p_^2^ = 0.115), indicating further exploration is warranted.

In order to determine if a subset of subjects are more impaired than most, the number of errors made (Fig 5E) and distance to escape (Fig 5F) were plotted individually against NAP scores for repeated ACHI subjects. This also allowed us to determine whether NAP scores may predict cognitive outcomes. In the female repeated ACHI group, moderate negative correlation was observed between the number of errors made and NAP score (**R**^**2**^ = 0.667), and the distance travelled to escape (**R**^**2**^ = 0.739). This was not present in the males, and had subsided in the females at PID 1 (**R**^**2**^ <0.1).

### ACHI did not produce significant motor impairments or increase anxiety-related behaviour

**Fig 6:**
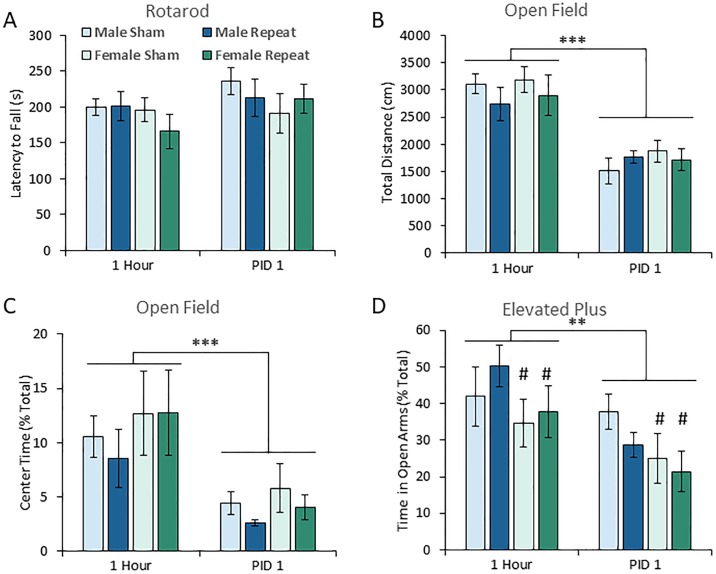
Repeat ACHI did not produce significant motor impairments or anxiety like behaviour at one hour and one day after injury. **(A)** There was no significant effect of injury, sex, or time point on the latency to fall from the Rotarod. Subjects were trained to use the Rotarod prior to injury, and then tested on a protocol accelerating from 10 to 50 RPM in a 5 minute interval. **(B)** Subjects were placed in a 100 cm diameter open field maze and allowed to explore freely for 5 minutes. Repeat ACHI did not impair overall mobility, as there were no significant differences in the total distance moved. A significant effect of post injury time point indicates all groups and sexes travelled a shorter distance on PID one. **(B)** There was no significant effect of sex or injury on the average proportion of time spent in the center (70cm diameter) of the open field maze, but all subjects spent significantly less time in the center on PID 1. **(D)** There was a significant effect of sex on the amount of time spent in the open arms of the elevated plus maze, indicating that across groups and time point’s females spent less time in the open arms. (**PID**: post injury day; ** p<0.01; *** p<0.001; **#** main effect of sex, p<0.05).

We used the Rotarod apparatus to examine learned motor skills after either single or repeated ACHI (n = 9 per group). All subjects were trained to the same criterion on the Rotarod prior to ACHI, and then tested after injury. The test protocol accelerated from a speed of 10 to 50 RPM in 5 minutes, and the latency to fall was recorded. As shown in (Fig 6A), there were no significant group, sex, or time point effects or interactions in Rotarod performance, indicating no motor impairment.

The total distance travelled by animals in an open field maze can also be used as a convergent means to assess motor function (Fig 6B). As is shown, there was no effect of ACHI on the total distance travelled in any group, providing further evidence that mobility was not impaired. Further support comes from the fact that the average speed, and percentage of time spent moving, in the Barnes maze, open field, end elevated plus maze was equivalent across groups (see S3 Fig). In all mazes, there were no significant injury effects or interactions on the average speed or time moving, confirming the absence of ACHI-induced motor impairment. Conversely, in the Barnes maze there was a significant effect of sex (F(1,32) = 4.296, p<0.046) on average speed, indicating that the females were moving significantly faster on both days, regardless of injury group.

The amount of time spent in the center (70cm diameter) of a round open field maze, and in the open arms of an elevated plus maze were used to examine signs of anxiety after ACHI (n = 9 per group). In the open field there were no significant injury group effects, sex effects, or interactions for the amount of time spent in the center (Fig 6C). Similarly, there were no significant injury group effects on time spent in the open arms in the elevated plus maze, indicating ACHI did not lead to acute elevations in anxiety (Fig 6D). There was a significant effect of sex (F(1,32) = 4.976, p = 0.021) meaning that females in all groups spent significantly less time in the open arms on both days, which is a putative indicator of anxiety. Conversely, the significant effect of time on open field distance (F(1,32) = 102.577, p<0.001); open field speed (F(1,32) = 102.577, p<0.001); open field time moving (F(1,32) = 102.577, p<0.001); and elevated plus maze time moving (F(1,32) = 102.577, p<0.001), provided evidence that normal changes in performance following multiple exposures to these mazes were occurring.

## Discussion

These initial experiments show that the ACHI model is a relatively simple high-throughput tool that reliably produces the key features of concussion outlined by the international consensus statement on concussion in sport [5]. In particular, we show that a single impact with this model can produce mild short-lived neurologic impairments that can be quantified using a simple NAP procedure. Repeated injuries produced more pronounced deficits including higher incidence and longer duration of LOC, acute neurologic impairment in NAP performance, and recall memory impairments in the Barnes maze. An *ex vivo* MRI analysis and cresyl violet histology indicated that these deficits occurred in the absence of any significant visible physical damage or structural changes in the brain. Moreover, NAP score impairment resolved spontaneously within 24 hours. Thus, our results indicate this model may be a useful tool to investigate the pathophysiology underlying repeated concussion in the developing brain.

A main advantage of the ACHI model and NAP scoring is that it allows the researcher to measure the rapid evolution of changes immediately after injury, without the potential confounds of surgical or anaesthetic recovery. Anaesthesia is known to have neuroprotective effects[39,40,66], and it is important to acknowledge that this has the potential to affect outcomes in models where it is used. A primary focus of future experiments is to explore whether acute anaesthetic exposure at the lower dosages commonly used in rodent models of mTBI may be a potential confounder.

Only a small subset of injured subjects lost consciousness, and these were predominantly subjects in the repeated ACHI group. These findings are in agreement with clinical observations, as well as those from similar models [5,9,10,41,67]. The observed LOC was comparable to that seen in high school and collegiate athletes, where only 5–9% of concussions produced LOC[9,10] with a median duration of 5 seconds, and with 91% regaining consciousness in less than 30 seconds[10].

A similar trend of graded symptom severity was observed in NAP findings where a repeated injury produced poorer performance than single ACHI. Overall, these results reflect a common trend in pre-clinical and clinical concussion research showing that repeated injuries tend to result in more severe neurologic impairments [18,42,63,68,69]. These data indicate our NAP score is a useful tool because it provides a simple, yet sensitive, assessment of neurologic function that can be rapidly applied in the diagnostically important time window immediately after the injury. However, it is limited in that it does not address more subtle cognitive, motor, and emotional changes that are common symptoms of clinical concussion [5,6,70–72].

Indeed, further behavioural assessment indicated that repeated ACHI produced acute recall memory impairments in the Barnes maze. Repeat injured subjects made significantly more errors compared to sham animals, indicating cognitive deficits. Conversely, no motor impairments or changes in anxiety-like behaviours were observed. Given that impairments after clinical concussion can emerge over time[5,73], and be transient and subtle[74], future work should extend the post-injury timeline and use more sensitive behavioural assessments to detect impairments after repeated ACHI. Future work should also explore how increasing the number of repeated injuries can produce more severe outcomes. This is emerging as an important consideration for many sports that involve the potential for head impact exposure. Recent attempts to quantify this in college athletes have indicated that football players experience an average of 6.3 impacts per practice, and 14.3 impacts per game[75], which is much greater than the four ACHI impacts administered here.

Behavioural outcomes can be compared to NAP score results to determine whether the NAP score may be predictive of the duration and complexity of symptoms in this model. Indeed, we found a moderate correlation between low NAP scores and poor Barnes maze performance in females. In clinical cases, the sport concussion assessment tool (SCAT) is a popular standardized tool used by physicians to help diagnose concussions. It is recommended for use immediately after a suspected concussion has occurred, and is also used to monitor recovery [14,76,77]. Initial SCAT performance may be predictive of the duration of recovery and severity of symptoms [78], and thus helpful for planning individualized treatment strategies. To that end, future work should aim to determine whether NAP score correlates with other impairments that may arise.

A major challenge in the diagnosis of clinical concussion is that symptoms often occur in the absence of visible damage on conventional neuroimaging (e.g., CT and structural MRI)[79–81]. Studies have noted changes in the volume of brain structures after concussion, but these changes are associated with a history of multiple head injuries, and occur after a long period of time [82]. We found that *ex vivo* whole bran scanning revealed no reduction in the volume of the hippocampus, cortex, or corpus callosum, indicating that single and repeated ACHI did not produce overt structural damage. This was confirmed by convergent cresyl violet histology in a separate cohort. Future studies will need to determine if multiple injuries at this early age produce long-term changes in brain development. This may require more advanced MRI methods, such as diffusion weighted MRI, to quantify indications of axonal or dendritic injury [83–86].

An important goal for clinical concussion research is to better understand the extensive heterogeneity in concussion symptoms and recovery. This has been linked to risk factors that were present before the injury, and the nature of the injury itself [87]. This problem can be investigated using models like ACHI by identifying pathophysiological differences between subsets of subjects that experience the most severe symptoms, and the ones that experience few or no symptoms, despite having received the same head impact. Investigating subtle physiologic differences between subjects that develop differing severities of symptoms in response to the same ACHI may provide insight on why symptomology is so diverse in clinical cases. The correlative comparison of NAP scores and Barnes maze outcomes revealed a subset of female subjects that performed much worse than their injured and uninjured counterparts alike. Some clinical studies have found that concussion symptoms in females tend to be more severe and longer in duration [18,88–91], although some experimental models find the opposite, supported by the neuroprotective effects of estrogen[92,93]. We failed to observe any significant injury induced differences between males and females in behavioural and structural outcomes. However, the subset of more severely impaired females suggests it is possible that sex differences in symptoms may require more sensitive tests to elucidate. Recently we have shown that measures of synaptic plasticity may help to elucidate functional sex differences following a closed head injury by a weight drop model in anaesthetised animals [94], and similar examinations may offer the veracity needed to adequately explore this issue following single and repeated ACHI.

We set out to design and characterise a model that produces mild closed head injury in juvenile rats that it is relevant to pediatric concussion. Taken together these observations reflect all four of the criteria that define a concussion outlined by the most recent consensus statement on concussion in sport [5]. We have shown that 1) an “impulsive” force transmitted to the head results in 2) the rapid onset of short-lived neurologic impairment that resolves spontaneously. This occurs 3) in the absence of overt structural changes, and 3) involves LOC and cognitive impairment in a small subset of cases. These results demonstrate the utility of the ACHI model for continuing pre-clinical research on concussions in the developing brain.

## Supporting information

S1 Fig Schematic diagram showing dimensions of custom 3D printed helmet to fit a juvenile (postnatal day 25–28) Long Evans rat. Helmets were 3D printed with 1.75mm ABS plastic filament using a MakerBot Replicator 2. They were placed over the rat’s head, and held in place with an elastic band under the chin and two sided tape.
(TIF)

S2 Fig The four tasks used in our modified NAP can individually distinguish between injury groups with varying success. **Startle response** was lost in one single injured subject, and in 45% of repeat injured subjects. **Limb extension** was impaired in 33% of single injured subjects, and in 60% of repeat injured subjects. **Beam walk** performance was impaired in 55% of repeat injured and 50% of single injured animals, but may have been more challenging overall as 10% of shams also failed. Similarly, 15% of sham controls failed to complete the **rotating beam** task, however injury-related impairment was evident as 22% of single- and 35% of repeat injured subjects also failed. Error bars show standard error of the proportion.
(TIF)

S3 Fig The average speed, and proportion of time moving in the Barnes, open field, and elevated plus mazes were recorded as secondary measures of general locomotion. There was a significant effect of sex on both speed, and percentage of time moving in the Barnes maze. There were no significant effects or interactions of group or sex in the open field and elevated plus maze. All groups travelled significantly slower and spent significantly less time moving in the open field on post injury day (PID) 1. Repeat injured males moved slower and spent less time moving on (PID 1) than 1 hour after final injury. (* p< 0.05; ** p< 0.01; *** p<0.001).
(TIF)

S1 Table This scoring guideline was used to assess the extent to which subjects resisted the soft plastic restraint used in the ACHI procedure. A low score of 0 indicates the subject entered the restraint without resisting, and remained silent and motionless while enclosed. Higher scores were assigned if the animal resisted entering the bag, and moved or vocalized after enclosed, depending on the severity. The maximum duration in the bag was limited to 5 minutes, and score of 4 or higher resulted in the animal being returned to their home cage to rest before trying again. The subject should be removed from the study if they continue to resist after three attempts. In this study, no animals reached the maximum duration of 5 minutes, and no animals were removed due to resisting restraint.
(TIF)

S2 Table Subjects were assessed for signs of pain and distress before and after each injury, and regularly during the week of recovery using. The upper portion of the table displays 5 categories of criteria that are common indicators of pain status in rodents. Each category is scored on a four point scale, where a 0 indicates no pain and a 3 indicates most severe pain. During an assessment, each animal is scored in each category. The lower portion of the table describes how to interpret the pain scale scores. It takes into account the total combined score from all categories, as well the highest score in a single category, in order to determine what action to take if an animal is showing signs of pain.
(TIF)

## References

[1] McCrory P, Feddermann-Demont N, Dvořák J, Cassidy JD, McIntosh A, Vos PE, et al What is the definition of sports-related concussion: a systematic review. Br J Sports Med. BMJ Publishing Group Ltd and British Association of Sport and Exercise Medicine; 2017;51: 877–887. doi: 10.1136/bjsports-2016-097393 29098981 10.1136/bjsports-2016-097393

[2] Langlois JA, Rutland-Brown W, Wald MM. The Epidemiology and Impact of Traumatic Brain Injury A Brief Overview. J Head Trauma Rehabil. 2006;21: 375–378. Available: https://insights.ovid.com/pubmed?pmid=16983222 16983222 10.1097/00001199-200609000-00001

[3] Harmon KG, Drezner JA, Gammons M, Guskiewicz KM, Halstead M, Herring SA, et al American Medical Society for Sports Medicine position statement: concussion in sport. Br J Sports Med. 2013;47: 15–26. doi: 10.1136/bjsports-2012-091941 23243113 10.1136/bjsports-2012-091941

[4] Daneshvar DH, Nowinski CJ, McKee AC, Cantu RC. The epidemiology of sport-related concussion. Clin Sports Med. 2011;30: 1–17, vii doi: 10.1016/j.csm.2010.08.006 21074078 10.1016/j.csm.2010.08.006 PMC2987636

[5] McCrory P, Meeuwisse WH, Aubry M, Cantu B, Dvořák J, Echemendia RJ, et al Consensus statement on concussion in sport—the 5 th international conference on concussion in sport held in Berlin, October 2016. Br J Sports Med. 2017;47: bjsports-2017-097699.

[6] Faul M, Wald MM, Xu L, Coronado VG. Traumatic brain injury in the United States; emergency department visits, hospitalizations, and deaths, 2002–2006 [Internet]. Atlanta (GA): Centers for Disease Control and Prevention, National Center for Injury Prevention and Control; 2010 https://stacks.cdc.gov/view/cdc/5571

[7] Stewart T Charyk, Gilliland J, Fraser DD. An epidemiologic profile of pediatric concussions: Identifying urban and rural differences. doi: 10.1097/TA.0b013e3182aafdf5 24553542 10.1097/TA.0b013e3182aafdf5

[8] Guskiewicz KM, Weaver NL, Padua DA, Garrett WE Jr. Epidemiology of Concussion in Collegiate and High School Football Players. Am J Sport Med. 2000;28: 643–650. Available: http://ajs.sagepub.com/cgi/content/short/28/5/643 10.1177/03635465000280050401 11032218

[9] Castile L, Collins CL, McIlvain NM, Comstock RD. The epidemiology of new versus recurrent sports concussions among high school athletes, 2005–2010. Br J Sports Med. 2012;46: 603–610. doi: 10.1136/bjsports-2011-090115 22144000 10.1136/bjsports-2011-090115

[10] Marshall SW, Guskiewicz KM, Shankar V, McCrea M, Cantu RC. Epidemiology of sports-related concussion in seven US high school and collegiate sports. Inj Epidemiol. 2015;2: 13 doi: 10.1186/s40621-015-0045-4 27747745 10.1186/s40621-015-0045-4 PMC5005709

[11] Teasdale G, Jennett B. ASSESSMENT OF COMA AND IMPAIRED CONSCIOUSNESSAssessment of coma and impaired consciousness: A Practical Scale. Lancet. 1974;304: 81–84. doi: 10.1016/S0140-6736(74)91639-0 10.1016/s0140-6736(74)91639-0 4136544

[12] McCrea M, Marshall SW, Cantu RC, Randolph C, Barr W, Onate JA, et al The NCAA Concussion Study. J Am Med Assoc. 2003;290: 2549–2555. 10.1001/jama.290.19.2549 14625331

[13] Hughes D, Jackson A, Mason D, Berry E, Hollis S, Yates D. Abnormalities on magnetic resonance imaging seen acutely following mild traumatic brain injury: correlation with neuropsychological tests and delayed recovery. Neuroradiology. Springer-Verlag; 2004;46: 550–558. doi: 10.1007/s00234-004-1227-x 15185054 10.1007/s00234-004-1227-x

[14] Echemendia RJ, Meeuwisse W, McCrory P, Davis GA, Putukian M, Leddy J, et al The sport concussion assessment tool 5th edition (SCAT5): Background and rationale. Br J Sports Med. 2017;51: 848–850. doi: 10.1136/bjsports-2017-097506 28446453 10.1136/bjsports-2017-097506

[15] Barlow KM, Crawford S, Stevenson A, Sandhu SS, Belanger F, Dewey D. Epidemiology of postconcussion syndrome in pediatric mild traumatic brain injury. Pediatrics. 2010;126: e374–e381. doi: 10.1542/peds.2009-0925 20660554 10.1542/peds.2009-0925

[16] Grubenhoff JA, Deakyne SJ, Brou L, Bajaj L, Comstock RD, Kirkwood MW. Acute concussion symptom severity and delayed symptom resolution. Pediatrics. 2014;134: 54–62. doi: 10.1542/peds.2013-2988 24958583 10.1542/peds.2013-2988

[17] Nelson LD, Guskiewicz KM, Barr WB, Hammeke TA, Randolph C, Ahn KW, et al Age differences in recovery after sport-related concussion: a comparison of high school and collegiate athletes. J Athl Train. National Athletic Trainers Association; 2016;51: 142–152. doi: 10.4085/1062-6050-51.4.04 26974186 10.4085/1062-6050-51.4.04 PMC4852320

[18] McCrory P, Meeuwisse WH, Aubry M, Cantu RC, Dvorák J, Echemendia RJ, et al Consensus statement on concussion in sport: the 4th International Conference on Concussion in Sport held in Zurich, November 2012. Br J Sports Med. 2013;47: 250–258. doi: 10.1136/bjsports-2013-092313 23479479 10.1136/bjsports-2013-092313

[19] Scopaz KA, Hatzenbuehler JR. Risk modifiers for concussion and prolonged recovery. Sports Health. SAGE Publications; 2013;5: 537–41. doi: 10.1177/1941738112473059 24427429 10.1177/1941738112473059 PMC3806172

[20] Zemper ED. Two-year prospective study of relative risk of a second cerebral concussion. Am J Phys Med Rehabil. 2003;82: 653–9. doi: 10.1097/01.PHM.0000083666.74494.BA 12960905 10.1097/01.PHM.0000083666.74494.BA

[21] Barkhoudarian G, Hovda DA, Giza CC. The molecular pathophysiology of concussive brain injury. Clinics in sports medicineClin Sport Med. 2011 pp. 33–48. doi: 10.1016/j.csm.2010.09.001 21074080 10.1016/j.csm.2010.09.001

[22] Tremblay S, De Beaumont L, Henry LC, Boulanger Y, Evans AC, Bourgouin P, et al Sports concussions and aging: a neuroimaging investigation. Cereb Cortex. 2013;23: 1159–66. doi: 10.1093/cercor/bhs102 22581847 10.1093/cercor/bhs102

[23] Bijur PE, Haslum M, Golding J. Cognitive outcomes of multiple mild head injuries in children. J Dev Behav Pediatr. 1996;17: 143–8. 8783059

[24] Matser JT, Kessels AGH, Jordan BD, Lezak MD, Troost J. Chronic traumatic brain injury in professional soccer players. Neurology. 1998;51: 791–796. doi: 10.1212/WNL.51.3.791 9748028 10.1212/wnl.51.3.791

[25] Wall SE, Williams WH, Cartwright-Hatton S, Kelly TP, Murray J, Murray M, et al Neuropsychological dysfunction following repeat concussions in jockeys. J Neurol Neurosurg Psychiatry. 2006;77: 518–20. doi: 10.1136/jnnp.2004.061044 16543534 10.1136/jnnp.2004.061044 PMC2077488

[26] Slobounov S, Slobounov E, Sebastianelli W, Cao C, Newell K. Differential rate of recovery in athletes after first and second concussion episodes. Neurosurgery. 2007;61: 338–44; discussion 344. doi: 10.1227/01.NEU.0000280001.03578.FF 17762746 10.1227/01.NEU.0000280001.03578.FF

[27] Gaetz M, Goodman D, Weinberg H. Electrophysiological evidence for the cumulative effects of concussion. Brain Inj. 2000;14: 1077–88. 11147580 10.1080/02699050050203577

[28] Pellman EJ, Viano DC, Tucker AM, Casson IR, Waeckerle JF. Concussion in professional football: reconstruction of game impacts and injuries. Neurosurgery. 2003;53: 799–812–4. 14519212 10.1093/neurosurgery/53.3.799

[29] Arciniegas DB, Anderson CA, Topkoff J, McAllister TW. Mild traumatic brain injury: a neuropsychiatric approach to diagnosis, evaluation, and treatment. Neuropsychiatr Dis Treat. 2005;1: 311–27. 18568112 PMC2424119

[30] Halstead ME, Walter KD. American Academy of Pediatrics. Clinical report—sport-related concussion in children and adolescents. Pediatrics. 2010;126: 597–615. doi: 10.1542/peds.2010-2005 20805152 10.1542/peds.2010-2005

[31] Guskiewicz KM, Marshall SW, Bailes J, McCrea M, Cantu RC, Randolph C, et al Association between recurrent concussion and late-life cognitive impairment in retired professional football players. Neurosurgery. 2005;57: 719–26-26. 16239884 10.1093/neurosurgery/57.4.719

[32] McKee AC, Cantu RC, Nowinski CJ, Hedley-Whyte ET, Gavett BE, Budson AE, et al Chronic traumatic encephalopathy in athletes: progressive tauopathy after repetitive head injury. J Neuropathol Exp Neurol. 2009;68: 709–35. doi: 10.1097/NEN.0b013e3181a9d503 19535999 10.1097/NEN.0b013e3181a9d503 PMC2945234

[33] Masel BE, DeWitt DS. Traumatic brain injury: a disease process, not an event. J Neurotrauma. 2010;27: 1529–1540. doi: 10.1089/neu.2010.1358 20504161 10.1089/neu.2010.1358

[34] Shultz SR, McDonald SJ, Vonder Haar C, Meconi A, Vink R, van Donkelaar P, et al The potential for animal models to provide insight into mild traumatic brain injury: Translational challenges and strategies. Neurosci Biobehav Rev. 2017;76: 396–414. doi: 10.1016/j.neubiorev.2016.09.014 27659125 10.1016/j.neubiorev.2016.09.014

[35] Xiong Y, Mahmood A, Chopp M. Animal models of traumatic brain injury. 2013;18: 1199–1216. 10.1038/nrn3407 PMC3951995 23329160

[36] Petraglia AL, Dashnaw ML, Turner RC, Bailes JE. Models of mild traumatic brain injury: translation of physiological and anatomic injury. Neurosurgery. 2014;75 Suppl 4: S34–49. doi: 10.1227/NEU.0000000000000472 25232883 10.1227/NEU.0000000000000472

[37] Giza CC, Hovda DA. The new neurometabolic cascade of concussion. Neurosurgery. 2014;75 Suppl 4: S24–33. doi: 10.1227/NEU.0000000000000505 25232881 10.1227/NEU.0000000000000505 PMC4479139

[38] Flower O, Hellings S. Sedation in Traumatic Brain Injury. Emerg Med Int. 2012;2012: 1–11. doi: 10.1155/2012/637171 23050154 10.1155/2012/637171 PMC3461283

[39] Statler KD, Alexander H, Vagni V, Dixon CE, Clark RSB, Jenkins L, et al Comparison of seven anesthetic agents on outcome after experimental traumatic brain injury in adult, male rats. J Neurotrauma. 2006;23: 97–108. doi: 10.1089/neu.2006.23.97 16430376 10.1089/neu.2006.23.97

[40] Statler KD, Alexander H, Vagni V, Holubkov R, Dixon CE, Clark RSB, et al Isoflurane exerts neuroprotective actions at or near the time of severe traumatic brain injury. Brain Res. 2006;1076: 216–24. doi: 10.1016/j.brainres.2005.12.106 16473332 10.1016/j.brainres.2005.12.106

[41] Petraglia A, Plog B, Walker C, Chen M, Czerniecka K, Dashnaw M, et al The pathophysiology underlying repetitive mild traumatic brain injury in a novel mouse model of chronic traumatic encephalopathy. Surg Neurol Int. 2014;5: 184 doi: 10.4103/2152-7806.147566 25593768 10.4103/2152-7806.147566 PMC4287910

[42] Petraglia AL, Plog BA, Dayawansa S, Chen M, Dashnaw ML, Czerniecka K, et al The spectrum of neurobehavioral sequelae after repetitive mild traumatic brain injury: a novel mouse model of chronic traumatic encephalopathy. J Neurotrauma. 2014;31: 1211–1224. doi: 10.1089/neu.2013.3255 24766454 10.1089/neu.2013.3255 PMC4082360

[43] Shohami E, Novikov M, Bass R. Long-term effect of HU-211, a novel non-competitive NMDA antagonist, on motor and memory functions after closed head injury in the rat. Brain Res. 1995;674: 55–62. doi: 10.1016/0006-8993(94)01433-I 7773695 10.1016/0006-8993(94)01433-i

[44] Ding J, Guo J, Yuan Q, Yuan F, Chen H, Tian H. Inhibition of phosphatase and tensin homolog deleted on chromosome 10 decreases rat cortical neuron injury and blood-brain barrier permeability, and improves neurological functional recovery in traumatic brain injury model. Borlongan C V., editor. PLoS One. 2013;8: e80429 doi: 10.1371/journal.pone.0080429 24312220 10.1371/journal.pone.0080429 PMC3842922

[45] Schaar KL, Brenneman MM, Savitz SI. Functional assessments in the rodent stroke model. Exp Transl Stroke Med. 2010;2: 13 doi: 10.1186/2040-7378-2-13 20642841 10.1186/2040-7378-2-13 PMC2915950

[46] Shapira Y, Shohami E, Sidi A, Soffer D, Freeman S, Cotev S. Experimental closed head injury in rats: mechanical, pathophysiologic, and neurologic properties. Crit Care Med. 1988;16: 258–265. 3277783 10.1097/00003246-198803000-00010

[47] Tustison NJ, Avants BB, Cook PA, Zheng Yuanjie, Egan A, Yushkevich PA, et al N4ITK: Improved N3 Bias Correction. IEEE Trans Med Imaging. 2010;29: 1310–1320. doi: 10.1109/TMI.2010.2046908 20378467 10.1109/TMI.2010.2046908 PMC3071855

[48] Avants BB, Tustison NJ, Song G, Cook PA, Klein A, Gee JC. A reproducible evaluation of ANTs similarity metric performance in brain image registration. Neuroimage. 2011;54: 2033–2044. doi: 10.1016/j.neuroimage.2010.09.025 20851191 10.1016/j.neuroimage.2010.09.025 PMC3065962

[49] Wright DK, Liu S, van der Poel C, McDonald SJ, Brady RD, Taylor L, et al Traumatic brain injury results in cellular, structural and functional changes resembling motor neuron disease. Cereb Cortex. San Diego, California: Elsevier Academic,; 2016;261: 412–421. doi: 10.1093/cercor/bhw254 27566977 10.1093/cercor/bhw254

[50] Wright DK, Trezise J, Kamnaksh A, Bekdash R, Johnston LA, Ordidge R, et al Behavioral, blood, and magnetic resonance imaging biomarkers of experimental mild traumatic brain injury. Sci Rep. 2016;6: 28713 doi: 10.1038/srep28713 27349514 10.1038/srep28713 PMC4923906

[51] Zhang Y, Brady M, Smith S. Segmentation of brain MR images through a hidden Markov random field model and the expectation-maximization algorithm. IEEE Trans Med Imaging. 2001;20: 45–57. doi: 10.1109/42.906424 11293691 10.1109/42.906424

[52] Johnstone VPA, Wright DK, Wong K, O’Brien TJ, Rajan R, Shultz SR. Experimental traumatic brain injury results in long-term recovery of functional responsiveness in sensory cortex but persisting structural changes and sensorimotor, cognitive, and emotional deficits. J Neurotrauma. 2015;32: 1333–1346. doi: 10.1089/neu.2014.3785 25739059 10.1089/neu.2014.3785

[53] Tan XL, Wright DK, Liu S, Hovens C, O’Brien TJ, Shultz SR. Sodium selenate, a protein phosphatase 2A activator, mitigates hyperphosphorylated tau and improves repeated mild traumatic brain injury outcomes. Neuropharmacology. 2016;108: 382–393. doi: 10.1016/j.neuropharm.2016.05.001 27163189 10.1016/j.neuropharm.2016.05.001

[54] Webster KM, Wright DK, Sun M, Semple BD, Ozturk E, Stein DG, et al Progesterone treatment reduces neuroinflammation, oxidative stress and brain damage and improves long-term outcomes in a rat model of repeated mild traumatic brain injury. J Neuroinflammation. 2015;12: 238 doi: 10.1186/s12974-015-0457-7 26683475 10.1186/s12974-015-0457-7 PMC4683966

[55] Rosenfeld CS, Ferguson SA. Barnes maze testing strategies with small and large rodent models. J Vis Exp. MyJoVE Corporation; 2014; e51194 doi: 10.3791/51194 24637673 10.3791/51194 PMC4140524

[56] Jones NC, Cardamone L, Williams JP, Salzberg MR, Myers D, O’Brien TJ. Experimental traumatic brain injury induces a pervasive hyperanxious phenotype in rats. J Neurotrauma. 2008 pp. 1367–1374. doi: 10.1089/neu.2008.0641 19061380 10.1089/neu.2008.0641

[57] Shultz SR, Wright DK, Zheng P, Stuchbery R, Liu SJ, Sashindranath M, et al Sodium selenate reduces hyperphosphorylated tau and improves outcomes after traumatic brain injury. Brain. 2015;138: 1297–1313. doi: 10.1093/brain/awv053 25771151 10.1093/brain/awv053 PMC5963409

[58] Prut L, Belzung C. The open field as a paradigm to measure the effects of drugs on anxiety-like behaviors: a review. Eur J Pharmacol. 2003;463: 3–33. 12600700 10.1016/s0014-2999(03)01272-x

[59] Jones NC, Salzberg MR, Kumar G, Couper A, Morris MJ, O’Brien TJ. Elevated anxiety and depressive-like behavior in a rat model of genetic generalized epilepsy suggesting common causation. Exp Neurol. 2008;209: 254–260. doi: 10.1016/j.expneurol.2007.09.026 18022621 10.1016/j.expneurol.2007.09.026

[60] Brocardo PS, Boehme F, Patten A, Cox A, Gil-Mohapel J, Christie BR. Anxiety- and depression-like behaviors are accompanied by an increase in oxidative stress in a rat model of fetal alcohol spectrum disorders: Protective effects of voluntary physical exercise. Neuropharmacology. 2012;62: 1607–18. doi: 10.1016/j.neuropharm.2011.10.006 22019722 10.1016/j.neuropharm.2011.10.006

[61] Hawley DF, Morch K, Christie BR, Leasure JL. Differential response of hippocampal subregions to stress and learning. Schmidt U, editor. PLoS One. 2012;7: e53126 doi: 10.1371/journal.pone.0053126 23285257 10.1371/journal.pone.0053126 PMC3532167

[62] Hsieh T-H, Kang J-W, Lai J-H, Huang Y-Z, Rotenberg A, Chen K-Y, et al Relationship of mechanical impact magnitude to neurologic dysfunction severity in a rat traumatic brain injury model. Borlongan CV, editor. PLoS One. 2017;12: e0178186 doi: 10.1371/journal.pone.0178186 28552947 10.1371/journal.pone.0178186 PMC5446124

[63] Shultz SR, Bao F, Omana V, Chiu C, Brown A, Cain DP. Repeated mild lateral fluid percussion brain injury in the rat causes cumulative long-term behavioral impairments, neuroinflammation, and cortical loss in an animal model of repeated concussion. J Neurotrauma. 2012;29: 281–294. doi: 10.1089/neu.2011.2123 21933013 10.1089/neu.2011.2123

[64] Luo Y, Zou H, Wu Y, Cai F, Zhang S, Song W. Mild traumatic brain injury induces memory deficits with alteration of gene expression profile. Sci Rep. 2017;7: 10846 doi: 10.1038/s41598-017-11458-9 28883638 10.1038/s41598-017-11458-9 PMC5589921

[65] Petraglia AL, Plog BA, Dayawansa S, Chen M, Dashnaw ML, Czerniecka K, et al The spectrum of neurobehavioral sequelae after repetitive mild traumatic brain injury: A novel mouse model of chronic traumatic encephalopathy. J Neurotrauma. 2014;31: 1211–1224. doi: 10.1089/neu.2013.3255 24766454 10.1089/neu.2013.3255 PMC4082360

[66] Hendrich KS, Kochanek PM, Melick JA, Schiding JK, Statler KD, Williams DS, et al Cerebral perfusion during anesthesia with fentanyl, isoflurane, or pentobarbital in normal rats studied by arterial spin-labeled MRI. Magn Reson Med. 2001;46: 202–6. 11443729 10.1002/mrm.1178

[67] Erlanger DM. Exposure to sub-concussive head injury in boxing and other sports. Brain Inj. 2015;29: 171–174. doi: 10.3109/02699052.2014.965211 25313457 10.3109/02699052.2014.965211

[68] Guskiewicz KM, McCrea M, Marshall SW, Cantu RC, Randolph C, Barr W, et al Cumulative effects associated with recurrent concussion in collegiate football players: the NCAA Concussion Study. JAMA. 2003;290: 2549–55. doi: 10.1001/jama.290.19.2549 14625331 10.1001/jama.290.19.2549

[69] McCrea M, Guskiewicz KM, Marshall SW, Barr W, Randolph C, Cantu RC, et al Acute effects and recovery time following concussion in collegiate football players: the NCAA Concussion Study. JAMA. 2003;290: 2556–2563. doi: 10.1001/jama.290.19.2556 14625332 10.1001/jama.290.19.2556

[70] Gibb R, Kolb B. A method for vibratome sectioning of Golgi-Cox stained whole rat brain. J Neurosci Methods. 1998;79: 1–4. 9531453 10.1016/s0165-0270(97)00163-5

[71] McCrea M, Iverson GL, McAllister TW, Hammeke Ta, Powell MR, Barr WB, et al An integrated review of recovery after mild traumatic brain injury (MTBI): implications for clinical management. Clin Neuropsychol. 2009;23: 1368–1390. doi: 10.1080/13854040903074652 19882476 10.1080/13854040903074652

[72] Carroll LJ, Cassidy JD, Peloso PM, Borg J, von Holst H, Holm L, et al Prognosis for mild traumatic brain injury: results of the WHO Collaborating Centre Task Force on Mild Traumatic Brain Injury. J Rehabil Med. 2004; 84–105. http://www.ncbi.nlm.nih.gov/pubmed/15083873 15083873 10.1080/16501960410023859

[73] Morgan CD, Zuckerman SL, Lee YM, King L, Beaird S, Sills AK, et al Predictors of postconcussion syndrome after sports-related concussion in young athletes: a matched case-control study. J Neurosurg Pediatr. 2015;15: 589–598. doi: 10.3171/2014.10.PEDS14356 25745949 10.3171/2014.10.PEDS14356

[74] Wright AD, Smirl JD, Bryk K, Fraser SK, Grewal HS, Jakovac M, et al Acute sport-related concussion induces transient impairment in dynamic cerebral auto regulation that is related to scat3 performance. Br J Sports Med. 2017;51: A38.1–A38. doi: 10.1136/bjsports-2016-097270.96

[75] Crisco JJ, Fiore R, Beckwith JG, Chu JJ, Brolinson PG, Duma S, et al Frequency and Location of Head Impact Exposures in Individual Collegiate Football Players. J Athl Train. 2010;45: 549–559. doi: 10.4085/1062-6050-45.6.549 21062178 10.4085/1062-6050-45.6.549 PMC2978006

[76] Chin EY, Nelson LD, Barr WB, McCrory P, McCrea MA. Reliability and validity of the sport concussion assessment tool-3 (scat3) in high school and collegiate athletes. Am J Sports Med. 2016;44: 2276–2285. doi: 10.1177/0363546516648141 27281276 10.1177/0363546516648141

[77] Davis GA, Purcell L, Schneider KJ, Yeates KO, Gioia GA, Anderson V, et al The child sport concussion assessment tool 5th edition (child scat5): background and rationale. Br J Sports Med. 2017;51: 859–861. Available: http://bjsm.bmj.com/content/51/11/859.abstract 28446452 10.1136/bjsports-2017-097492

[78] Tkachenko N, Singh K, Hasanaj L, Serrano L, Kothare SV. Sleep disorders associated with mild traumatic brain injury using sport concussion assessment tool 3. Pediatr Neurol. 2016;57: 46–50.e1. doi: 10.1016/j.pediatrneurol.2015.12.019 26795630 10.1016/j.pediatrneurol.2015.12.019

[79] Lee H, Wintermark M, Gean AD, Ghajar J, Manley GT, Mukherjee P. Focal lesions in acute mild traumatic brain injury and neurocognitive outcome: CT versus 3T MRI. J Neurotrauma. 2008;25: 1049–1056. doi: 10.1089/neu.2008.0566 18707244 10.1089/neu.2008.0566

[80] Chiara Ricciardi M, Bokkers RPH, Butman JA, Hammoud DA, Pham DL, Warach S, et al Trauma-specific brain abnormalities in suspected mild traumatic brain injury patients identified in the first 48 hours after injury: a blinded magnetic resonance imaging comparative study including suspected acute minor stroke patients. J Neurotrauma. 2017;34: 23–30. doi: 10.1089/neu.2015.4338 27215444 10.1089/neu.2015.4338 PMC5198056

[81] Clough M, Mutimer S, Wright DK, Tsang A, Costello DM, Gardner AJ, et al Oculomotor cognitive control abnormalities in australian rules football players with a history of concussion. J Neurotrauma. 2018; neu.2017.5204. doi: 10.1089/neu.2017.5204 29228862 10.1089/neu.2017.5204

[82] List J, Ott S, Bukowski M, Lindenberg R, Flöel A. Cognitive function and brain structure after recurrent mild traumatic brain injuries in young-to-middle-aged adults. Front Hum Neurosci. Frontiers; 2015;9: 228 doi: 10.3389/fnhum.2015.00228 26052275 10.3389/fnhum.2015.00228 PMC4440350

[83] Dimou S, Lagopoulos J. Toward objective markers of concussion in sport: a review of white matter and neurometabolic changes in the brain after sports-related concussion. J Neurotrauma. 2014;31: 413–24. doi: 10.1089/neu.2013.3050 24266534 10.1089/neu.2013.3050

[84] Dodd AB, Epstein K, Ling JM, Mayer AR. Diffusion tensor imaging findings in semi-acute mild traumatic brain injury. Mary Ann Liebert Inc.; 2014 pp. 1235–1248. doi: 10.1089/neu.2014.3337 24779720 10.1089/neu.2014.3337

[85] Wilde EA, Bouix S, Tate DF, Lin AP, Newsome MR, Taylor BA, et al Advanced neuroimaging applied to veterans and service personnel with traumatic brain injury: state of the art and potential benefits. Brain Imaging Behav. Springer US; 2015;9: 367–402. doi: 10.1007/s11682-015-9444-y 26350144 10.1007/s11682-015-9444-y PMC6547383

[86] Xiong K lin, Zhu Y shan, Zhang W guo. Diffusion tensor imaging and magnetic resonance spectroscopy in traumatic brain injury: a review of recent literature. Brain Imaging Behav Springer New York LLC; 2014;8: 487–496. doi: 10.1007/s11682-013-9288-2 24449140 10.1007/s11682-013-9288-2

[87] Rosenbaum SB, Lipton ML. Embracing chaos: the scope and importance of clinical and pathological heterogeneity in mTBI. Brain Imaging Behav. 2012;6: 255–282. doi: 10.1007/s11682-012-9162-7 22549452 10.1007/s11682-012-9162-7

[88] Bazarian JJ, Blyth B, Mookerjee S, He H, McDermott MP. Sex differences in outcome after mild traumatic brain injury. J Neurotrauma. 2010;27: 527–539. doi: 10.1089/neu.2009.1068 19938945 10.1089/neu.2009.1068 PMC2867588

[89] Covassin T, Elbin RJ, Harris W, Parker T, Kontos A. The role of age and sex in symptoms, neurocognitive performance, and postural stability in athletes after concussion. Am J Sports Med. 2012;40: 1303–12. doi: 10.1177/0363546512444554 22539534 10.1177/0363546512444554

[90] Covassin T, Moran R, Elbin RJ. Sex Differences in Reported Concussion Injury Rates and Time Loss From Participation: An Update of the National Collegiate Athletic Association Injury Surveillance Program From 2004–2005 Through 2008–2009. J Athl Train. 2016;51: 189–194. doi: 10.4085/1062-6050-51.3.05 26950073 10.4085/1062-6050-51.3.05 PMC4852524

[91] Covassin T, Elbin RJ, Larson E, Kontos AP. Sex and age differences in depression and baseline sport-related concussion neurocognitive performance and symptoms. Clin J Sport Med. 2012;22: 98–104. doi: 10.1097/JSM.0b013e31823403d2 22246342 10.1097/JSM.0b013e31823403d2

[92] Bramlett HM, Dietrich WD. neuropathological protection after traumatic brain injury in intact female rats versus males or ovariectomized females. J Neurotrauma. 2001;18: 891–900. doi: 10.1089/089771501750451811 11565601 10.1089/089771501750451811

[93] Roof RL, Hall ED. Gender differences in acute CNS trauma and stroke: neuroprotective effects of estrogen and progesterone. J Neurotrauma. 2000;17: 367–88. doi: 10.1089/neu.2000.17.367 10833057 10.1089/neu.2000.17.367

[94] White ER, Pinar C, Bostrom CA, Meconi A, Christie BR. Mild traumatic brain injury produces long-lasting deficits in synaptic plasticity in the female juvenile hippocampus. J Neurotrauma. 2017;34: 1111–1123. doi: 10.1089/neu.2016.4638 27735217 10.1089/neu.2016.4638

