# Supplementary figures and images for: Repeated mild traumatic brain injury can cause acute neurologic impairment without overt structural damage in juvenile rats
Source: PLoS One. 2018 May 8;13(5):e0197187. doi: 10.1371/journal.pone.0197187 (PMC5940222; doi:10.1371/journal.pone.0197187)

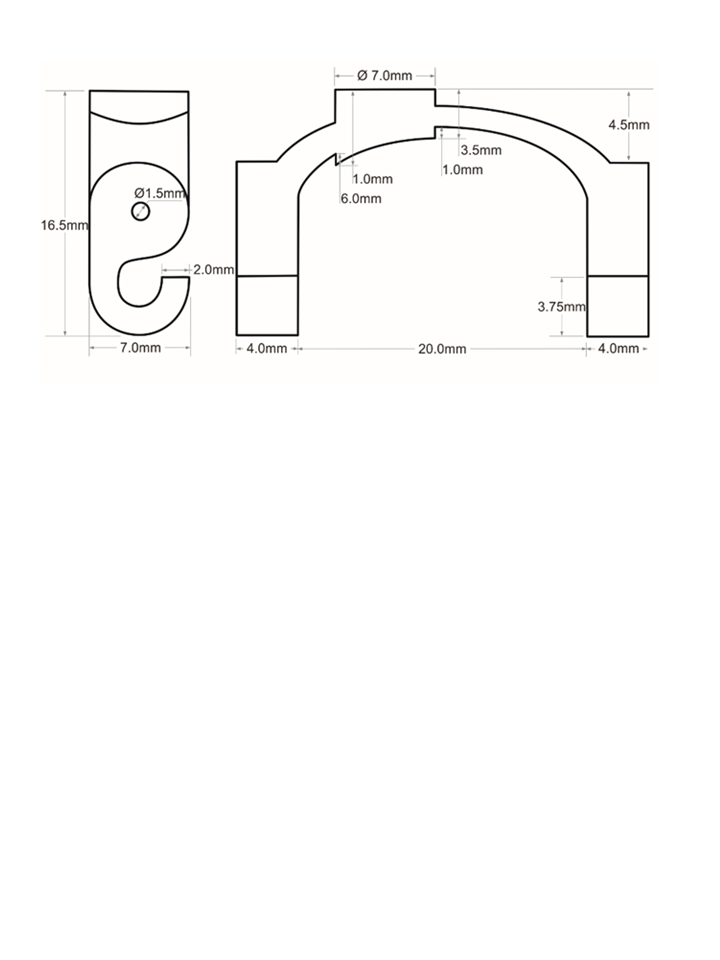

Supplement: S1 Fig — Helmets were 3D printed with 1.75mm ABS plastic filament using a MakerBot Replicator 2. They were placed over the rat’s head, and held in place with an elastic band under the chin and two sided tape. (TIF) [file pone.0197187.s001.TIF]

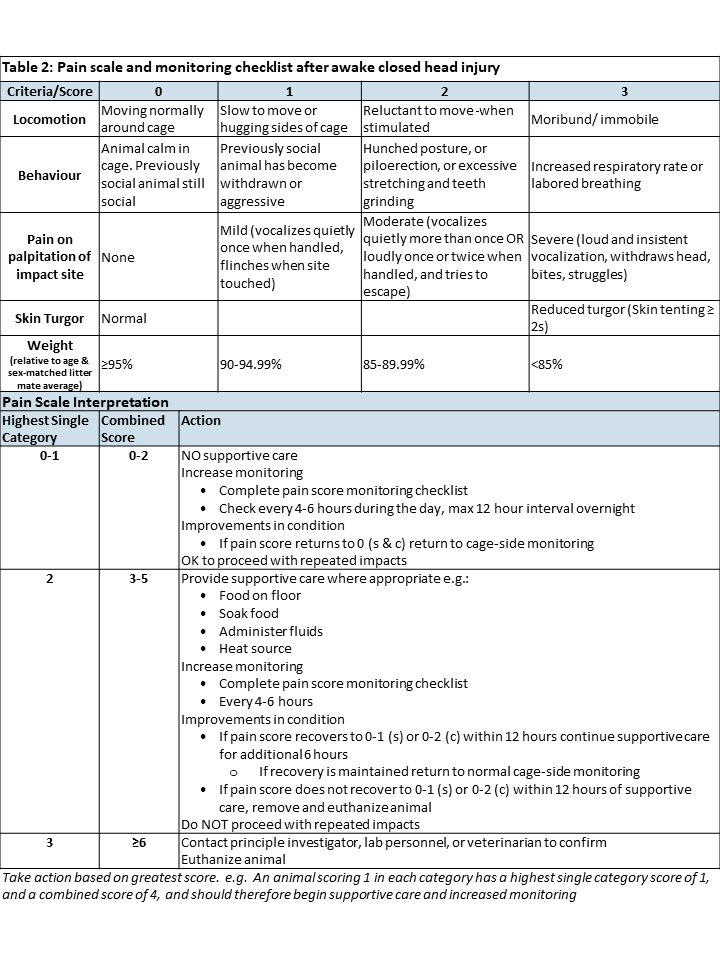

Supplement: S2 Fig — Startle response was lost in one single injured subject, and in 45% of repeat injured subjects. Limb extension was impaired in 33% of single injured subjects, and in 60% of repeat injured subjects. Beam walk performance was impaired in 55% of repeat injured and 50% of single injured animals, but may have been more challenging overall as 10% of shams also failed. Similarly, 15% of sham controls failed to complete the rotating beam task, however injury-related impairment was evident as 22% of single- and 35% of repeat injured subjects also failed. Error bars show standard error of the proportion. (TIF) [file pone.0197187.s002.TIF]

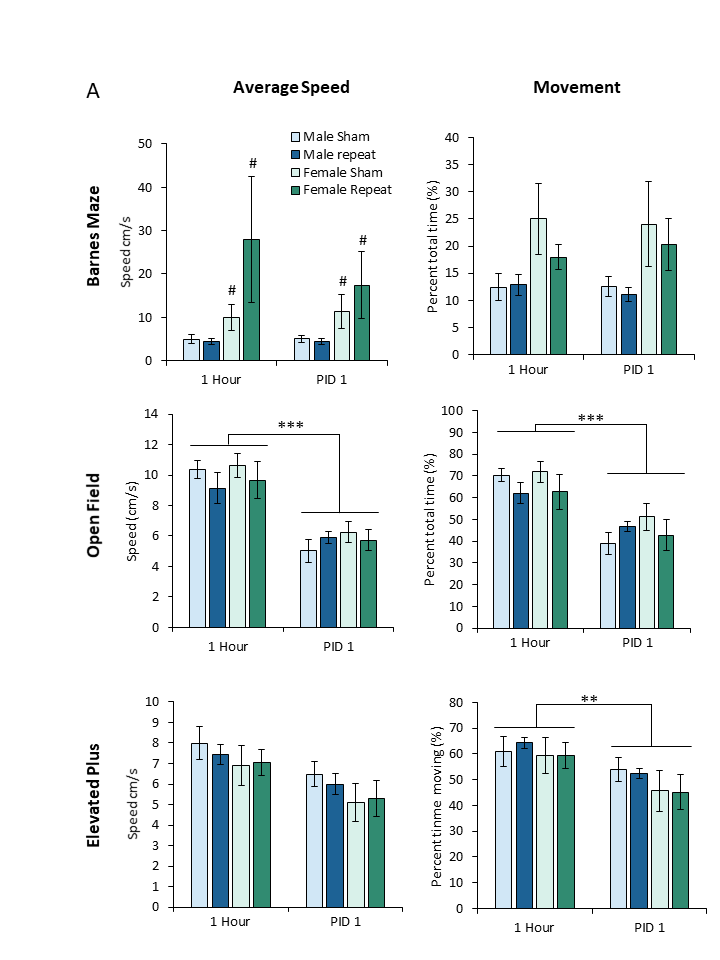

Supplement: S3 Fig — There was a significant effect of sex on both speed, and percentage of time moving in the Barnes maze. There were no significant effects or interactions of group or sex in the open field and elevated plus maze. All groups travelled significantly slower and spent significantly less time moving in the open field on post injury day (PID) 1. Repeat injured males moved slower and spent less time moving on (PID 1) than 1 hour after final injury. (* p< 0.05; ** p< 0.01; *** p<0.001). (TIF) [file pone.0197187.s003.TIF]

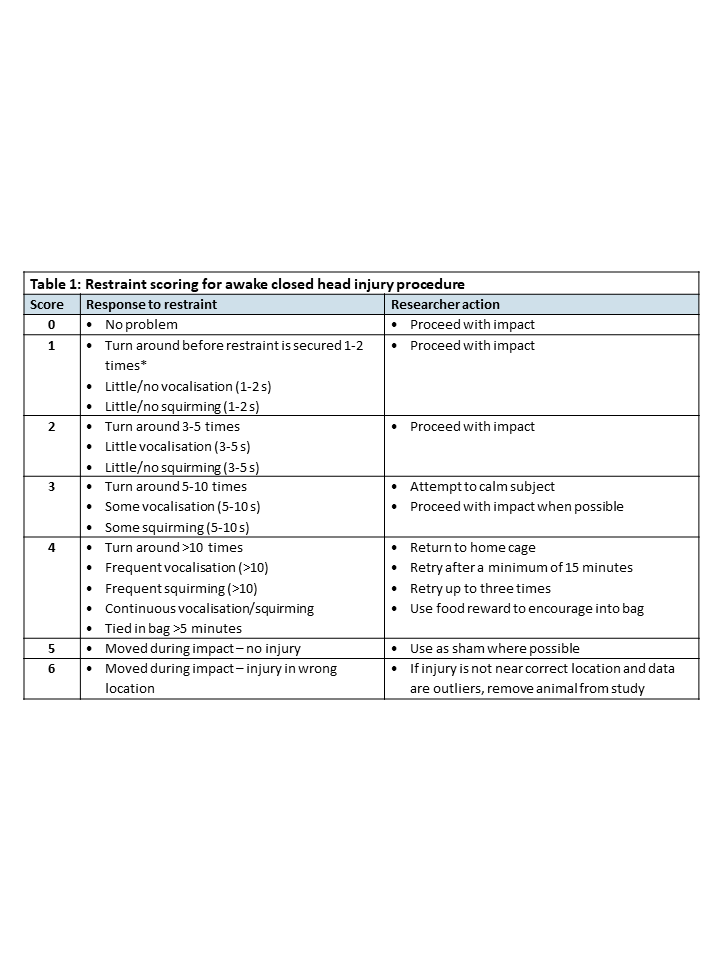

Supplement: S1 Table — A low score of 0 indicates the subject entered the restraint without resisting, and remained silent and motionless while enclosed. Higher scores were assigned if the animal resisted entering the bag, and moved or vocalized after enclosed, depending on the severity. The maximum duration in the bag was limited to 5 minutes, and score of 4 or higher resulted in the animal being returned to their home cage to rest before trying again. The subject should be removed from the study if they continue to resist after three attempts. In this study, no animals reached the maximum duration of 5 minutes, and no animals were removed due to resisting restraint. (TIF) [file pone.0197187.s004.TIF]

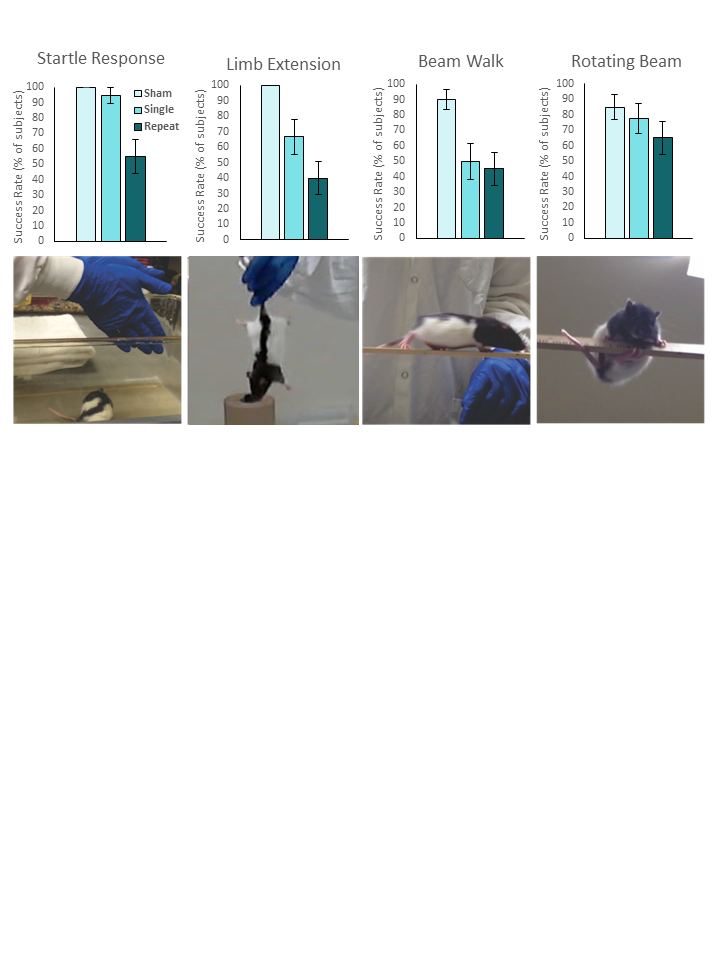

Supplement: S2 Table — The upper portion of the table displays 5 categories of criteria that are common indicators of pain status in rodents. Each category is scored on a four point scale, where a 0 indicates no pain and a 3 indicates most severe pain. During an assessment, each animal is scored in each category. The lower portion of the table describes how to interpret the pain scale scores. It takes into account the total combined score from all categories, as well the highest score in a single category, in order to determine what action to take if an animal is showing signs of pain. (TIF) [file pone.0197187.s005.TIF]
